# Pneumoperitoneum-induced pneumothorax during laparoscopic living donor hepatectomy: a case report

**DOI:** 10.1186/s12893-020-00868-8

**Published:** 2020-09-16

**Authors:** Min Suk Chae, Jueun Kwak, Kyungmoon Roh, Minhee Kim, Sungeun Park, Ho Joong Choi, Jaesik Park, Jung-Woo Shim, Hyung Mook Lee, Yong-Suk Kim, Young Eun Moon, Sang Hyun Hong

**Affiliations:** 1 Department of Anesthesiology and Pain Medicine, Seoul St. Mary’s Hospital, College of Medicine, The Catholic University of Korea, 222, Banpo-daero, Seocho-gu, Seoul, 06591 Republic of Korea; 2 Department of Anesthesiology and Pain Medicin, Yeouido St. Mary’s Hospital, College of Medicine, The Catholic University of Korea, Seoul, Republic of Korea; 3 Department of Anesthesiology and Pain Medicine, Eunpyeong St. Mary’s Hospital, College of Medicine, The Catholic University of Korea, Seoul, Republic of Korea; 4 Department of Anesthesiology and Pain Medicine, Bucheon St. Mary’s Hospital, College of Medicine, The Catholic University of Korea, Seoul, Republic of Korea; 5 Department of Surgery, Seoul St. Mary’s Hospital, College of Medicine, The Catholic University of Korea, Seoul, Republic of Korea

**Keywords:** Pneumothorax, Laparoscopic surgery, Living donor, Liver transplantation, Lung ultrasound

## Abstract

**Background:**

We present a living donor case with an unexpected large-volume pneumothorax diagnosed using lung ultrasound during a laparoscopic hepatectomy for liver transplantation (LT).

**Case presentation:**

A 38-year-old healthy female living donor underwent elective laparoscopic right hepatectomy. The preoperative chest radiography (CXR) and computed tomography images were normal. The surgery was uneventfully performed with tolerable CO_2_ insufflation and the head-up position. SpO_2_ decreased and airway peak pressure increased abruptly after beginning the surgery. There were no improvements in the SpO_2_ or airway pressure despite adjusting the endotracheal tube. Eventually, lung ultrasound was performed to rule out a pneumothorax, and we verified the stratosphere sign as a marker for the pneumothorax. The surgeon was asked to temporarily hold the surgery and cease with the pneumoperitoneum. Portable CXR verified a large right pneumothorax with a small degree of left lung collapse; thus, a chest tube was inserted on the right side. The hemodynamic parameters fully recovered and were stable, and the surgery continued laparoscopically. The surgeon explored the diaphragm and surrounding structures to detect any defects or injuries, but there were no abnormal findings. The postoperative course was uneventful, and a follow-up CXR revealed complete resolution of the two-sided pneumothorax.

**Conclusion:**

This living donor case suggests that a pneumothorax can occur during laparoscopic hepatectomy due to the escape of intraperitoneal CO_2_ gas into the pleural cavity. Because missing the chance to identify a pneumothorax early significantly decreases the safety for living donors, point-of-care lung ultrasound may help attending physicians reach the final diagnosis of an intraoperative pneumothorax more rapidly and to plan the treatment more effectively.

## Background

Laparoscopic donor surgery for liver transplantation (LT) reduces postoperative complications, such as surgical site infection and postoperative ascites, and also allows for a shorter hospital stay and earlier return to normal activities [1]. Therefore, it is ideal for living donors who have lived a healthy life, and its adoption is rapidly increasing [2]. A laparoscopic donor hepatectomy is performed through CO_2_ insufflation of the abdominal cavity to achieve an optimal surgical view. However, laparoscopic surgery may cause severe CO_2_-related complications, such as pneumothorax, pneumoperitoneum, and subcutaneous emphysema [3]. Although the incidence of pneumothorax is low during laparoscopy-based surgery, the complication can be severe and life-threatening [4].

Chest radiography (CXR) and computed tomography (CT) have largely been used as lung imaging tools to diagnose abnormal lung conditions in the emergency and critical care settings. However, the pitfalls of CXR are technical difficulties leading to limited accuracy and exposure of the patients to radiation [5, 6]. There is an added need to mobilize patients for CT and expose them to higher radiation, in addition to poor repeatability and high cost [7, 8]. A superior imaging option is lung ultrasound, which has many advantages, including easy bedside accessibility and reproducibility without invasive and radiative features [9].

Here, we present a living donor case with an unexpected large-volume CO_2_ pneumothorax diagnosed using point-of-care lung ultrasound during laparoscopic hepatectomy for liver LT.

## Case presentation

A 38-year-old female living donor (height 162.3 cm; weight 53.5 kg; body mass index 20.3 kg/m^2^) was scheduled for elective laparoscopic right hepatectomy for LT. She was clinically acceptable for donation surgery according to multidisciplinary assessments based on living liver donation guidelines [10, 11]. She had no history of diabetes mellitus, hypertension, cerebro- and cardiovascular diseases, hepatitis or pneumothorax, and there were nonspecific physical findings. The CXR and chest CT image findings were nonspecific, and there was no evidence of fatty liver or focal hepatic lesions in abdominal CT images. Sinus rhythm was recorded on an electrocardiogram. A central venous catheter (Arrow 7 Fr catheter; Teleflex, Wayne, PA, USA) was implemented to administer intravenous fluids and medications without complications in the right internal jugular vein under sonographic guidance, and the patency of catheterization was confirmed by CXR. Her laboratory findings were as follows: hemoglobin, 12.0 g/dL; white blood cell count, 6.94 × 10^9^/L (neutrophils, 57.2%); platelet count, 271,000/μL; creatinine, 0.64 mg/dL; albumin, 4.2 g/dL; aspartate and alanine aminotransferase, 19 U/L and 11 U/L, respectively; total bilirubin, 0.39 mg/dL; and prothrombin time, 111.6% (INR: 0.94).

Balanced anesthesia was induced with 100 mg propofol (Fresenius Kabi, Bad Homburg, Germany) and 50 mg rocuronium (Merck Sharp & Dohme Corp., Kenilworth, NJ, USA), and was maintained with desflurane (Baxter, Deerfield, IL, USA) in oxygen/medical air (FiO_2_ 50%) with volume-controlled mechanical ventilation (tidal volume: 400 mL; respiration rate: 12/min; inspiration and expiration ratio: 1:2). A neuromuscular block was maintained with rocuronium. Her vital signs (i.e., systolic [SBP] and diastolic [DBP] blood pressure; heart rate [HR]; and body temperature) and hypnotic depth (bispectral index monitor; Medtronic, Minneapolis, MN, USA) were continuously monitored and managed, as appropriate (Table 1).

**Table 1:** Serial changes in hemodynamic parameters during laparoscopic donor hepatectomy

		Laparoscopic surgery							
		Beginning surgery			+ 30 min	+ 45 min	+ 60 min	+ 240 min	End of surgery
	Anesthetic induction	Trocar insertion	CO_2_ insufflation	Head-up position	Lung ultrasound^a^	CXR	Chest tube insertion	Hepatic artery clamping	Follow-up CXR
SBP (mmHg)	111	91	104	101	82	104	147	106	123
DBP (mmHg)	76	49	63	65	41	68	98	67	59
HR (beats/min)	80	72	78	80	110	81	90	81	78
BIS	40	42	45	50	46	45	45	44	44
BT (°C)	36.5	36.5	36.4	36.4	36.3	36.2	36.2	36.1	36
APP (mmH_2_O)	15	15	18	17	38	25	15	17	16
TV (mL)	400	400	380	410	180	310	400	400	400
SpO_2_ (%)	99	100	98	100	88	92	100	100	100
ETCO_2_ (mmHg)	30	30	35	34	48	38	35	35	30

^**a**^Cessation of CO_2_ gas insufflation

*Abbreviation*: *CXR* Chest X-ray, *SBP* Systolic blood pressure, *DBP* Diastolic blood pressure, *HR* Heart rate, *BIS* Bispectral index, *BT* Body temperature, *APP* Airway peak pressure, *TV* Tidal volume, *SpO*_*2*_ Oxygen saturation, *ETCO*_*2*_ End tidal CO_2_

A laparoscopic hepatectomy was performed using the five-trocar technique and the trocars were placed uneventfully. Abdominal CO_2_ insufflation was performed with a pressure of 10–12 mmHg. The patient was placed in a 30° head-up position to assist with surgical exposure. The harmonic scalpel (Ethicon Inc., Cincinnati, OH, USA), with transaction and hemostasis of the liver around tissues, was meticulously applied for liver mobilization.

**Fig. 1:**
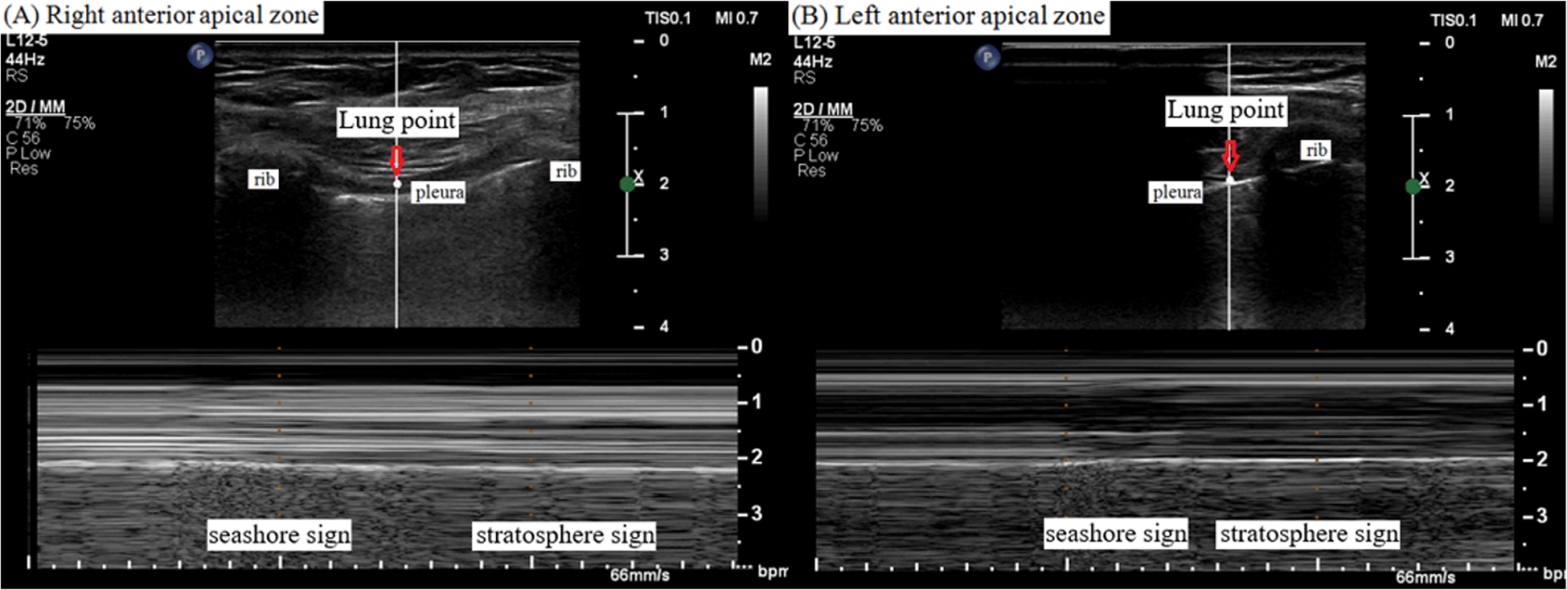
Lung point on point-of-care lung ultrasound. **a** Right anterior apical zone. **b** Left anterior apical zone. Arrows indicate the lung point where the normal lung pattern (i.e., seashore sign) replaces the pneumothorax pattern (i.e., stratosphere sign) with inspiration

**Fig. 2:**
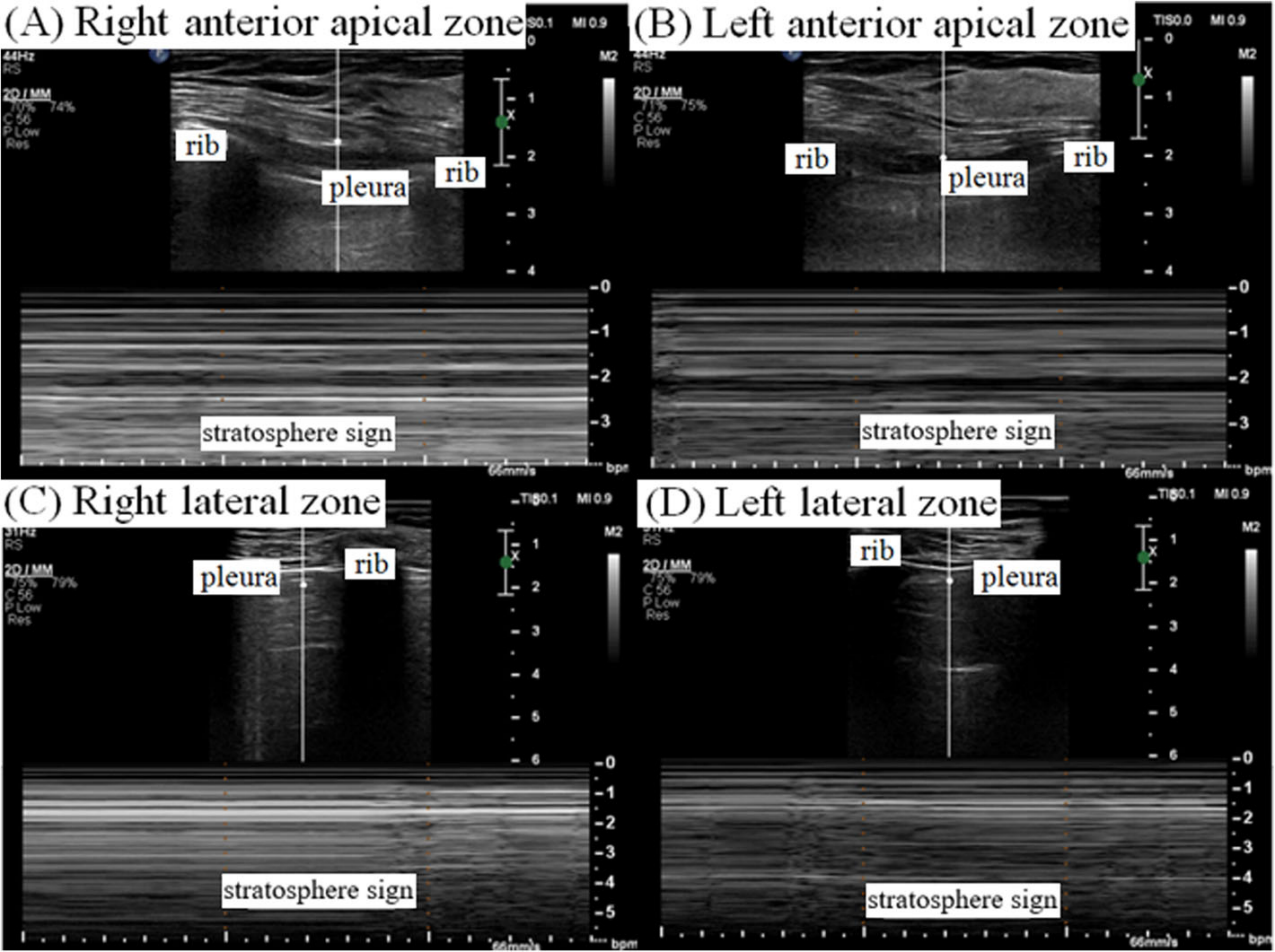
Stratosphere sign on point-of-care lung ultrasound. **a** Right anterior apical zone. **b** Left anterior apical zone. **c** Right lateral zone. **d** Left lateral zone

**Fig. 3:**
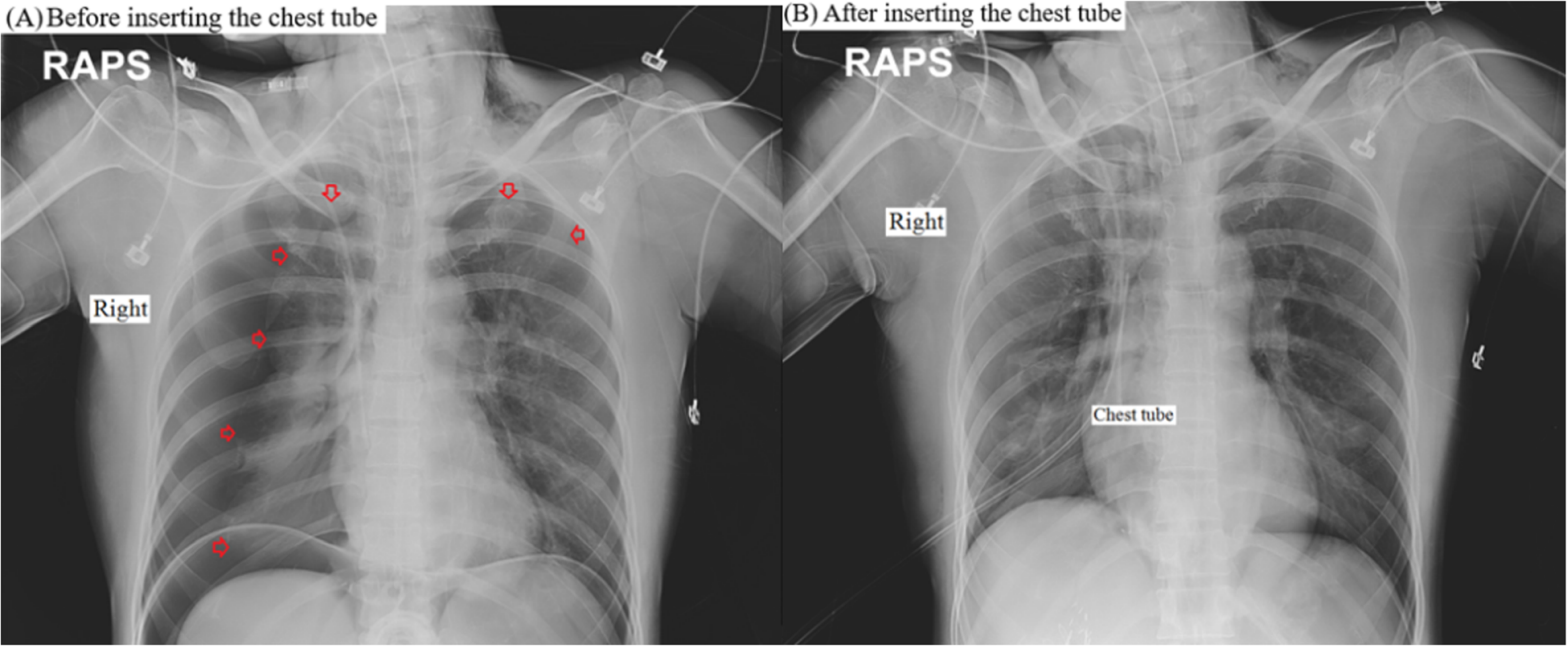
Chest radiography. **a** Before inserting the chest tube. **b** After inserting the chest tube. Arrows indicate the visceral pleural line with the absence of vascular marking beyond the pleural line

**Fig. 4:**
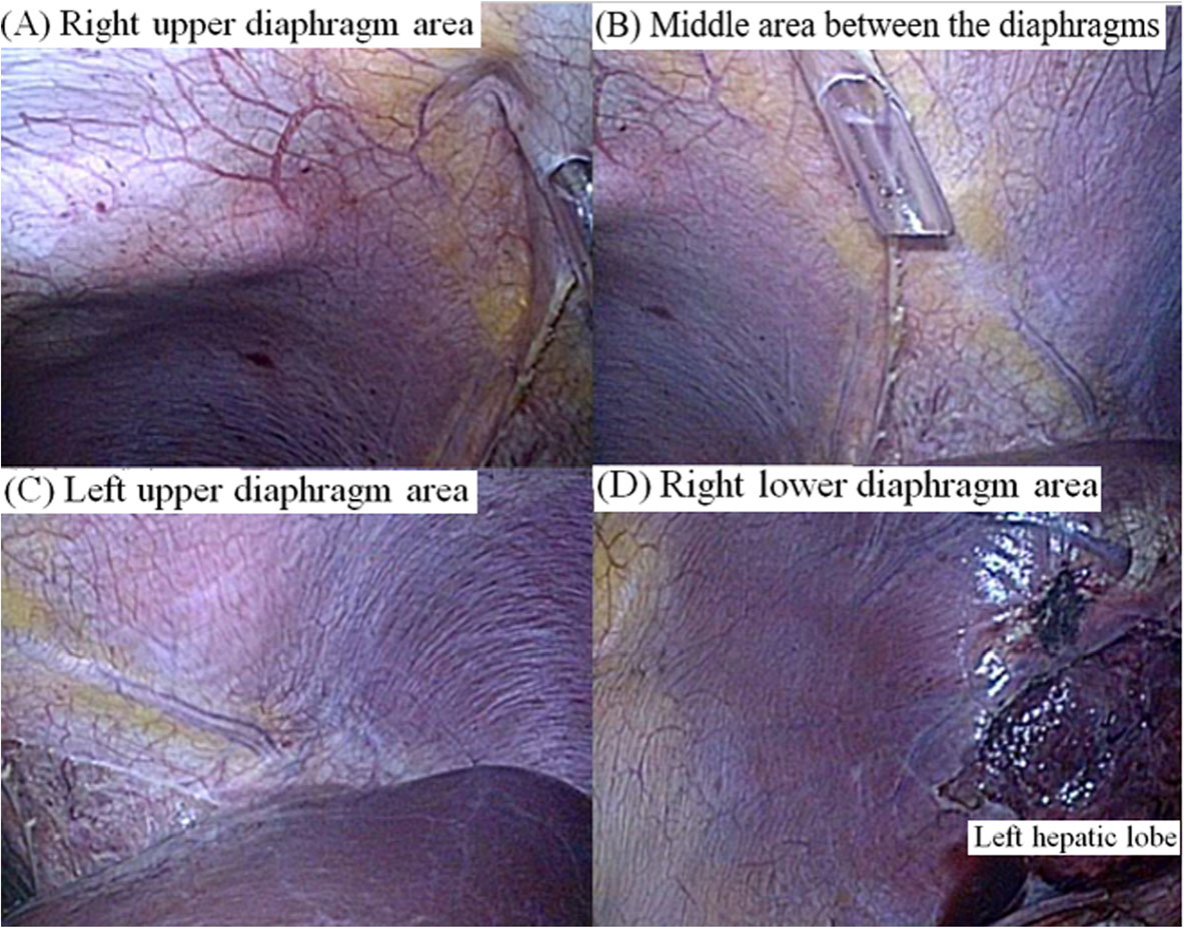
Endoscopic diaphragmatic view. **a** Right upper diaphragm area. **b** Middle area between the diaphragms. **c** Left upper diaphragm area. **d** Right lower diaphragm area

Saturation of peripheral oxygen (SpO_2_) decreased to 90%, and airway peak pressure reached 35 cmH_2_O 30 min after beginning the laparoscopic surgery. Chest auscultation revealed normal breath sounds over the left chest but reduced breath sounds on the right. Because we assumed that this could be due to kinking or migration of the endotracheal tube or due to obstructive secretions in the endotracheal tube, we carefully checked the tube and smoothly toileted inside the tube using a fiberoptic bronchoscope. Chest auscultation and movement seemed to recover during inspiration and expiration. However, airway peak pressure abruptly reached 38 cmH_2_O again and tidal volume simultaneously decreased to 180 mL. Her hemodynamic parameters began to change as SpO_2_ was 88%, end-tidal CO_2_ increased to 48 mmHg, SBP/DBP decreased to 82/41, and HR reached 110 beats/min. As a result, a sonographic examination of the lungs (Affiniti 70C ultrasound system; Philips, Amsterdam, the Netherlands) was performed to rule out pneumothorax, but we verified the lung points as a sonographic marker for occult pneumothorax in both anterior apical zones (Fig. 1). A pneumothorax was suspected, and the surgeon was asked to temporarily pause the surgery and cease gas insufflation. Lung ultrasound using a sterile drape was meticulously performed in the anterior apical zone (i.e., the mid-clavicular line at the second to third intercostal space) and lateral zone (i.e., the lateral-clavicular line at lateral and slightly superior to the nipple) with the patient in the supine position, and the stratosphere sign was identified as a critical marker for pneumothorax (Fig. 2). Portable CXR verified a right-dominant large pneumothorax with a small degree of left lung collapse; thus, a chest tube was inserted on the right side without complications (Fig. 3). After passive decompression using a chest tube, her hemodynamic parameters fully recovered, and the surgery continued laparoscopically with the chest tube inserted and close monitoring of the left-side lung field using lung ultrasound and respiratory monitoring. The surgeon explored both the diaphragm and surrounding structures to detect any defects or injuries, but there were no specific abnormal findings (Fig. 4). The surgery was completed uneventfully, and after manual deflation of the abdomen, an alveolar recruitment maneuver (i.e., manual inflation of 30 cmH_2_O for 30 s) was performed several times. We confirmed the return of both equal breath sounds, and the donor was extubated and transported to the postanesthesia care unit.

**Fig. 5:**
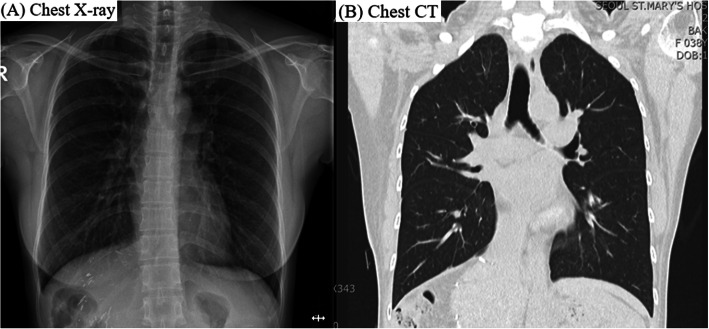
**a** Chest radiography and **b** computed tomography (CT) at hospital discharge

The postoperative recovery course was uneventful, and follow-up CXR and CT images revealed complete resolution of the two-sided pneumothorax (Fig. 5). She was discharged on postoperative day 5 without any complications.

## Discussion

The main lessons of this case are that anesthesiologists and surgeons should promptly and accurately diagnose and manage a pneumothorax that may threaten living donor safety, such as a tension pneumothorax. During laparoscopic liver donation surgery, point-of-care lung ultrasound was an accessible and reproducible diagnostic tool for the pneumothorax with the ‘stratosphere sign’ [9, 12]. Early identification of the pneumothorax using point-of-care lung ultrasound led to timely management of the pneumothorax during surgery without fatal complications, and our donor was discharged without sequela.

Although pneumothorax, as a complication of laparoscopic surgery, is rare, several cases have been reported [13–16]. A study by Pizzo et al. [4] suggested that in 1765 patients undergoing laparoscopic renal surgery, 10 patients (0.6%) intraoperatively experienced pleural injury that subsequently led to pneumothorax; they involved inadvertent trocar placement (2 patients) during splenic (2 patients), liver (2 patients), or ascending colon (1 patient) mobilization, and dissection of the upper renal pole (2 patients) and of a large renal cyst off of the diaphragm (1 patient). A study by Murdock et al. [16] suggested that in 968 patients undergoing laparoscopy-based surgery, the incidence of pneumothorax/pneumomediastinum was 1.9% and was markedly related to prolonged surgical manipulation and a large amount or pressure of CO_2_ gas insufflation.

An intraoperative pneumothorax may largely originate from iatrogenic procedures and/or congenital defects. The iatrogenic causes include barotrauma and rupture of emphysematous bullae or a bleb due to mechanical ventilation, direct injury to the diaphragm during surgical dissection, central line malpractice, or errant trocar implementation [17–19]. A CO_2_ pneumothorax can be caused by congenital defects in the diaphragm or other defects/fistulas around sites where the aorta, vena cava, and esophagus traverse the diaphragm [20–22]. In addition, various intraoperative factors, including a higher ETCO_2_ level (i.e., ≥50 mmHg), prolonged operation duration (i.e., ≥200 min), and rapid and higher CO_2_ insufflation pressure, may account for a pneumothorax [17, 23]. A CO_2_ pneumothorax is more likely to occur on the right side during hepatic side surgery, such as a laparoscopic cholecystectomy, because of surgical exposure of the omentum, which naturally covers a diaphragmatic defect or weak point [24, 25]. The surgical heads-up position with CO_2_ gas insufflation may increase the risk for pneumothorax because the position may push the liver and omentum downward and eventually result in exposure of a diaphragmatic defect in the pressurized abdominal cavity. In our case, it was difficult to diagnose the pneumothorax intraoperatively and to decide whether it was suitable to perform invasive therapies, such as implemented thoracocentesis or placing a chest tube because the surgical and anesthetic practices were uneventful; CO_2_ gas insufflation pressure was acceptable, and our living donor was young and healthy. However, a diaphragmatic defect cannot be excluded and suprahepatic surgical exploration may injure the diaphragm by accident. CO_2_ gas can also dissect into the pleural space along the vena cava. Additionally, because pathologic findings of the chest, including bullae or a macro-shunt, were not found in preoperative chest CT images, a micro-channel of iatrogenic or congenital origin between the abdominal and chest cavities may have been largely responsible for insufflation of the CO_2_ gas into the right lung field and subsequently the occurrence of pneumothorax. Although breath sounds and chest wall motion of both lung fields seemed to be normal during manual Ambu-bagging, the respiratory signs of pneumothorax, such as increases in ETCO_2_ and airway pressure and a decrease in SpO_2_, gradually developed during the CO_2_ pneumoperitoneum, and severity worsened as the surgery progressed. We performed a sonographic assessment and detected a CO_2_ pneumothorax without pulmonary origin that stopped CO_2_ gas insufflation and prevented hemodynamic aggravation.

The absence of lung sliding, where air separates the visceral and parietal pleurae, is demonstrated via M-mode ultrasonography as a linear pattern from superficial tissue to the pleural line and a similar pattern from the pleural line to deep tissue known as the ‘stratosphere (barcode) sign or lung sliding sign’. This sonographic finding highly suggests a pneumothorax (i.e., 95% sensitivity; 100% negative predictive value; and 87% positive predictive value) [26, 27]. The point where the normal lung pattern (i.e., seashore sign) replaces the pneumothorax pattern (i.e., stratosphere sign) with inspiration, is known as the ‘lung point,’ which significantly suggests occult or partial pneumothorax [28]. In addition, lung ultrasound is more efficient and better for diagnosing a pneumothorax in emergency cases than bedside CXR [28]. The time spent diagnosing a pneumothorax can be reduced using lung ultrasound compared to CXR [29]. Although lung CT has been established as the gold standard for diagnosing pathological lung conditions, including a pneumothorax, patients undergoing surgery in an operating room have limited use of CT [30]. In our case, lung ultrasound provided accurate evidence for the pneumothorax, and appropriately guided intraoperative donor care, including avoiding a pneumoperitoneum and performing the chest tube drainage. Based on practical guidelines recommending that the ultrasound examination should systematically cover both lung fields which are divided into six regions, such as upper and lower parts of the anterior, lateral and posterior chest wall using anterior- and posterior-axillary lines as anatomical landmarks, in the supine position setting [9, 12, 30], we examined both lung fields and successfully verified the barcode sign during surgery. Considering the relationship between the position of a surgical patient and air collection sites, the sonographic probe should be properly located in the operating room. In addition, close communication and collaboration between surgeons and anesthesiologists over surgical, hemodynamic, and sonographic findings are essential to minimize the risk for a fatal prognosis.

This living donor case suggests that a pneumothorax can unexpectedly occur during laparoscopic hepatectomy due to the escape of intraperitoneal CO_2_ gas into the pleural cavity through a potential micro-channel of iatrogenic or congenital origin between the abdomen and chest. Although CO_2_ pneumothorax is well tolerated and rapidly reversed after the release of the pneumoperitoneum, unlike a pneumothorax secondary to lung trauma [13, 31, 32], missing the chance to identify a pneumothorax early significantly decreases safety for living donors. Point-of-care lung ultrasound is considered a better imaging technique than CXR and an alternative imaging technique to CT during laparoscopic donor hepatectomy, and it will help attending physicians reach the final diagnosis of intraoperative pneumothorax more rapidly and plan treatments more effectively.

## Supplementary information

**Additional file 1.**

## Data Availability

All data and materials described in the manuscript will be freely available for non-commercial purposes.

## Abbreviations

LT: Liver transplantatio
CXR: Chest radiography
CT: Computed tomography
SBP: Systolic blood pressure
DBP: Diastolic blood pressure
HR: Heart rate
SpO_2_: Saturation of peripheral oxygen
ETCO_2_: End tidal CO_2_
