# Supplementary material for: Pneumoperitoneum-induced pneumothorax during laparoscopic living donor hepatectomy: a case report
Source: BMC Surg. 2020 Sep 16;20:206. doi: 10.1186/s12893-020-00868-8 (PMC7495872; doi:10.1186/s12893-020-00868-8)
Supplement: Supplementary file 1 — Additional file 1. [file 12893_2020_868_MOESM1_ESM.docx]

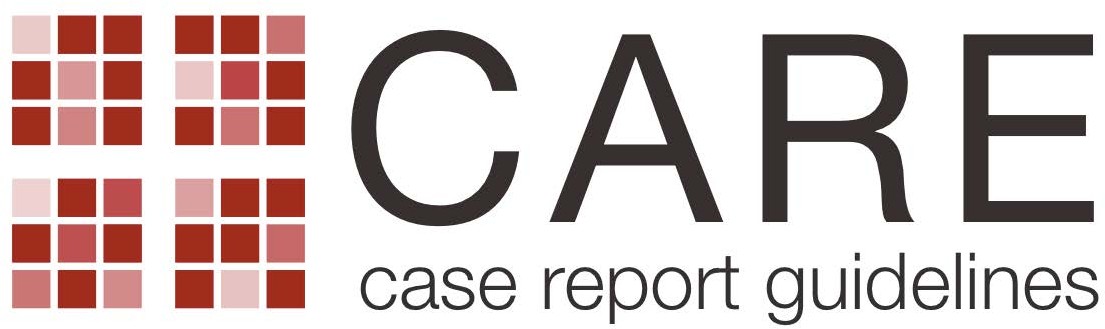
CARE Checklist of information to include when writing a case report
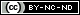


**Topic Item Checklist item description Reported on Line**

**Title 1** The diagnosis or intervention of primary focus followed by the words “case report” page 1

**Key Words 2** 2 to 5 key words that identify diagnoses or interventions in this case report, including "case report" page 3

# Abstract

**(no references)**

**3a** Introduction: What is unique about this case and what does it add to the scientific literature? Page 3

**3b** Main symptoms and/or important clinical findings Page 3

**3c** The main diagnoses, therapeutic interventions, and outcomes Page 3

**3d** Conclusion—What is the main “take-away” lesson(s) from this case? Page 3

**Introduction 4** One or two paragraphs summarizing why this case is unique (**may include references**) Page 4

**Patient Information 5a** De-identified patient specific information Page 5 - 7

**5b** Primary concerns and symptoms of the patient Page 5 - 7

**5c** Medical, family, and psycho-social history including relevant genetic information Page 5 - 7

**5d** Relevant past interventions with outcomes Page 5 - 7

# Clinical Findings

**Timeline**

**Diagnostic Assessment**

**Therapeutic Intervention**

**Follow-up and Outcomes**

1. Describe significant physical examination (PE) and important clinical findings Page 5 - 7
2. Historical and current information from this episode of care organized as a timeline Page 5 - 7

**8a** Diagnostic testing (such as PE, laboratory testing, imaging, surveys). Page 5 - 7

**8b** Diagnostic challenges (such as access to testing, financial, or cultural) Page 5 - 7

**8c** Diagnosis (including other diagnoses considered) Page 5 - 7

**8d** Prognosis (such as staging in oncology) where applicable Page 5 - 7

**9a** Types of therapeutic intervention (such as pharmacologic, surgical, preventive, self-care) Page 5 - 7

**9b** Administration of therapeutic intervention (such as dosage, strength, duration) Page 5 - 7

**9c** Changes in therapeutic intervention (with rationale) Page 5 - 7

**10a** Clinician and patient-assessed outcomes (if available) Page 5 - 7

**10b** Important follow-up diagnostic and other test results Page 5 - 7

**10c** Intervention adherence and tolerability (How was this assessed?) Page 5 - 7

**10d** Adverse and unanticipated events Page 5 - 7

**Discussion 11a** A scientific discussion of the strengths AND limitations associated with this case report Page 7 - 10

**11b** Discussion of the relevant medical literature **with references** Page 7 - 10

**11c** The scientific rationale for any conclusions (including assessment of possible causes) Page 7 - 10

**11d** The primary “take-away” lessons of this case report (without references) in a one paragraph conclusion Page 7 - 10

**Patient Perspective 12** The patient should share their perspective in one to two paragraphs on the treatment(s) they received Page 7 - 10

**Informed Consent 13** Did the patient give informed consent? Please provide if requested . . . . . . . . . . . . . . . . . . . . . . . . . . . . . . . . . . . . . . **Yes V No**
